# The Importance of Population Growth and Regulation in Human Life History Evolution

**DOI:** 10.1371/journal.pone.0119789

**Published:** 2015-04-01

**Authors:** Ryan Baldini

**Affiliations:** Department of Anthropology. Stanford University, Stanford, CA 94305; University College London, UNITED KINGDOM

## Abstract

Explaining the evolution of human life history traits remains an important challenge for evolutionary anthropologists. Progress is hindered by a poor appreciation of how demographic factors affect the action of natural selection. I review life history theory showing that the quantity maximized by selection depends on whether and how population growth is regulated. I show that the common use of *R*, a strategy’s expected lifetime number of offspring, as a fitness maximand is only appropriate under a strict set of conditions, which are apparently unappreciated by anthropologists. To concretely show how demography-free life history theory can lead to errors, I reanalyze an influential model of human life history evolution, which investigated the coevolution of a long lifespan and late age of maturity. I show that the model’s conclusions do not hold under simple changes to the implicitly assumed mechanism of density dependence, even when stated assumptions remain unchanged. This analysis suggests that progress in human life history theory requires better understanding of the demography of our ancestors.

## Introduction

The unusual life history characteristics of humans provide a unique challenge to evolutionary anthropologists. Humans have a distinctive life history even among our closest living relatives: compared to other primates, we endure a long juvenile period of intensive growth, learning, and extreme dependency; we mature and reproduce late; we have long reproductive careers with short interbirth intervals; and we enjoy a long post-reproductive lifespan [1, 2]. This pronounced life course has attracted the attention of many theorists, and consequently some novel, human-specific theories have been proposed [1, 3, 4, 5] alongside more species-general life history theory [2, 6, 7].

Theorists often use optimization methods to understand how selection acts on life histories. The general method is to maximize a fitness function with respect to some physiological tradeoff. For example, we might assume that energy devoted to immune function cannot be devoted to gamete production (or any other biological function). A life history theorist might ask: which proportion of energy allocation to immune function will serve to maximize fitness? The maximization method is widely used in introductory life history textbooks on which empirical researchers strongly rely (e.g., [8, 9, 10]).

One of the major difficulties with optimization methods arises in the first step: that of specifying the fitness quantity to be maximized. As I demonstrate here, this problem is usually solved by specifying a complete model of demography and inheritance, from which one can *derive* a quantity which natural selection maximizes. Unfortunately, traditional life history theory often does not begin with the construction of complete population models, but rather with the theorist simply choosing a common fitness quantity. The chosen maximand is usually only valid under particular demographic conditions [11, 12], but, without full justification from a population model, most readers never know these conditions.

This paper aims to clarify the role of demography in life history theory, with a focus on how demographic assumptions determine the correct fitness maximand. First, I review two general models of selection on age-structured populations, showing which quantities are maximized under density-independent and density-dependent population growth. I then show that one common maximand, a strategy’s expected lifetime number of offspring (often denoted by *R*), is valid under only a limited set of conditions. These conditions do not appear to be widely appreciated among anthropologists. In particular, zero population growth alone is not sufficient to justify *R* as a fitness maximand. Then, to concretely show how demographically vague models can lead to premature conclusions, I reanalyze the model in an influential paper on the evolution of human life histories: Kaplan et al.’s [1] “A theory of human life history: diet, intelligence, and longevity.” I show that Kaplan et al.’s conclusions, which address the coevolution of a long lifespan and late age of maturity, do not hold under simple changes in the action of density-dependence, even while their stated assumptions remain intact. This sensitivity to the precise form of population regulation implies that we need to better understand the demographic history and prehistory of our species if our models are to provide specific, realistic predictions.

## What does selection maximize?

Here I review how different forms of population regulation lead to different maximization procedures. There are an infinite number of possible demographic scenarios one could assume, but much can be learned from studying the two broad categories of density-independent or -dependent growth. The latter usually implies that population growth rates decrease as the population becomes more dense, via increased competition for resources. This section borrows heavily from Charlesworth’s classic book, *Evolution in Age-Structured Populations*[13]. To simplify matters, I assume asexual, haploid genetic inheritance and no frequency-dependent selection, as is often done in fitness maximization models. The extension to sexual reproduction is usually straightforward [13]. The goal, for each case, is to find the quantity that natural selection maximizes, and to briefly explain how a theorist can apply the results to specific problems.

### Maximization under density-independent growth

First, consider a population of individuals whose survival and reproduction is not affected by population density. Specifically, the probability of surviving to age *x* is *l*(*x*), and the average number of offspring produced by an individual of age *x* is *m*(*x*). Under some fairly lenient demographic assumptions [14], such a population will approach growth rate of *r*, which is found by solving the equation

$$\begin{array}{c} 1=\int_{0}^{\infty }e^{-rx}\;l\left(x\right)m\left(x\right)dx. \end{array}$$ (1)

Now suppose a new allele arises in the population, whose life history is characterized by the survival and fertility functions *l* ^′^ and *m* ^′^. Will this new allele increase in frequency relative to the common one? One can show mathematically that it will spread only if its growth rate, *r* ^′^, is greater than that of the common allele [13], i.e.,

$$\begin{array}{c} r^{'}>r \end{array}$$ (2)

Thus, we conclude that *selection maximizes the population growth rate under density independence*. This result is fairly intuitive. With asexual reproduction and density independence, strategies can be imagined as entirely separate populations, each growing at a different exponential rate. The one that grows fastest approaches frequency 1, relative to all others. With sexual reproduction, the result is similar: a rare allele will spread only if the growth rate of heterozygotes is greater than that of the resident population [13]. A stochastic analog to *r*, usually denoted *a*, is used when environments vary over time, such that *l* and *m* are not fixed [15].

It follows from this result that selection does not necessarily maximize the expected lifetime number of offspring, *R*, under density-independent growth. This fact is not immediately obvious, so I provide a simple demonstration. Imagine two asexual life history strategies, *A* and *B*. All individuals survive for exactly two seasons and produce two offspring, but *A* and *B* differ in their timing of reproduction. Individuals of strategy *A* produce one offspring in both their first and second seasons, while individuals of strategy *B* produce both offspring in their second season only.

Table 1 shows the result of selection, beginning with a population composed of one newborn individual of each strategy. Strategy *A* grows faster than *B* and eventually approaches frequency 1 in the population. The reason is that *A* reproduces much earlier than *B*, and thus has a shorter generation length and ultimately a higher *r*.

**Table 1:** *N*_*A*_ and *N*_*B*_ give the population sizes for strategies *A* and *B*, respectively. *p*_*A*_ is the frequency of strategy *A* among the total population. The frequency of *p* fluctuates as it approaches 1 because strategy *B* does not converge a unique age distribution and growth rate.

Season	0	1	2	3	4	5	6	7	8	9	10	20	30
*N*_*A*_	1	2	3	5	8	13	21	34	55	89	144	1.8e4	2.2e6
*N*_*B*_	1	1	2	2	4	4	8	8	16	16	32	1.0e3	3.3e4
*p*_*A*_	0.5	0.67	0.6	0.71	0.67	0.76	0.72	0.81	0.77	0.85	0.82	0.95	0.99

Overall, these results readily imply a maximization methodology: we simply write down *l* and *m* as a function of some set of tradeoffs, find *r* by solving Equation (1), and then maximize *r* with respect to the tradeoff. In practice, this can be challenging, as the integral in Equation (1) often cannot be solved in elementary terms. In these cases, numerical approximations can be used.

### Maximization under density-dependent growth

Density-dependent population growth, under which vital rates depend on population density, greatly complicates analysis of selection. There are an infinite number of possible functional forms for density dependence, and so general results are difficult to discover. In particular, density dependence can cause population growth cycles and chaos, which probably preclude maximization principles. Combining stochastic environmental variation with density dependence can also preclude maximization [16]. Charlesworth [13] found one useful case under which maximization does occur, and I will treat this case throughout. Consider a population whose survival and fertility rates, *l* and *m*, are decreasing functions of the population size *N*, because increased population density augments competition for resources. (Actually, Charlesworth [13] shows that *N* could be some increasing function of the number of individuals in various age group. We might call this quantity the *critical population size*; it could be the total population size, the total number of reproductive females, the sum of individuals weighted by their size, etc.) Assume also that the population eventually approaches a stable equilibrium value $\hat{N}$, which can be considered the population’s carrying capacity. $\hat{N}$ is found by solving the equation

$$\begin{array}{c} 1=\int_{x=0}^{\infty }l\left(x,\hat{N}\right)m\left(x,\hat{N}\right)dx. \end{array}$$ (3)

Note that *l* (the survival function) and *m* (the fertility function) are now written as functions of the population size.

Now imagine, again, that a new allele arises that alters the survival and fertility functions. Charlesworth [13] shows that the frequency of this allele increases only if the equilibrium population size of the new strategy, ${\hat{N}}^{\prime }$, is greater than that of the common strategy, i.e.,

$$\begin{array}{c} {\hat{N}}^{'}>\hat{N}. \end{array}$$ (4)

We conclude that *selection maximizes population size under density dependence* (or, more generally, the critical population size). This result can be understood as follows. Suppose a resident strategy has achieved equilibrium such that *N*, the critical population size, has approach $\hat{N}$. Now suppose a new strategy arises in the population, which is characterized by a slightly larger ${\hat{N}}^{\prime }$—that is, if it were to grow by itself in a separate environment, it would achieve higher *N* at equlibrium than the resident strategy. Since *l* and *m* are decreasing functions of *N*, and $\hat{N}<{\hat{N}}^{\prime }$, it follows that the new strategy will grow upon introduction. This pushes the population above the original strategy’s carrying capacity, causing the original strategy to shrink. Eventually the new strategy replaces the old entirely, ultimately resulting in a larger equilibrium population size.

The general procedure, then, is to specify some tradeoffs in *l* and *m*, use Equation (3) to solve for $\hat{N}$, and then maximize $\hat{N}$. In practice, there is no limit to how complex the functions *l* and *m* can be with respect to population size, so this procedure can be very difficult.

## When is *R* an appropriate maximand?

The expected lifetime number of offspring, commonly denoted by *R*, is often used as a maximand in life history theory. Hawkes et al.’s model [3], for example, relies heavily on the theory developed by Charnov (e.g. [17]), which is based on *R* as a maximand. Kaplan et al.’s model [1], which I reanalyze below, uses *R* to develop a general theory of human life history evolution. *R* is commonly used outside of anthropology; many examples can be found in [8, 9] and [10]. An important problem, then, is to determine the conditions under which *R* can be used in optimality models. I show here that the conditions are more limiting than some authors appear to appreciate.

It is frequently claimed that *R* is a correct fitness measure under zero population growth. This is true in the sense that it determines the rate of gene frequency change over a short period when *r* = 0 [13]. As such, *R* is an appropriate *dynamical* measure of fitness under such conditions. In particular, *R* is a useful dynamical measure of fitness in density-dependent populations because, over evolutionary time, the population will usually be near carrying capacity at which *r* = 0 (assuming no cyclic or chaotic dynamics). Maximization methods, however, treat long-term evolutionary questions: they assume that enough time has passed for all possible strategies to invade, and for selection to bring the optimal strategy to high frequency (see [18] for a discussion of timescale in models of natural selection). As a fitness *maximand*, *R* generally fails, even in density-dependent populations with zero growth. The reason is that, under density dependence, a strategy does not have a single number *R*. Rather, *R* becomes a *function* of population size. Without a single number by which to compare different strategies, one cannot use a maximization procedure. One could choose an arbitrary *N* at which to evaluate *R*, but this has no justification in general—especially because the ordering of *R* across strategies may vary with *N*. In fact, the theory of the previous section shows that the single number of interest is $\hat{N}$, a strategy’s carrying capacity.

The preceding logic suggests that maximizing *R* would be correct if the relative ordering of *R* across all strategies is independent of *N*. Then one could pick any arbitrary population size *N* and maximize *R*(*N*); the resulting strategy would necessarily have the largest carrying capacity and therefore would be optimal. Under what assumptions does this condition hold? Mathematically, it holds if every strategy’s *R* is equally affected by density via a single multiplier that is independent of age among reproductive age classes (see just below). Biologically, this holds under the following cases of density dependence:

1. Density dependence only affects expected fertility via the same multiplier for all strategies, independently of age. Survival is not density-dependent.
2. Density dependence only affects the probability of survival to reproductive ages via the same multiplier for all strategies. Fertility is not density-dependent.
3. Density dependence affects both fertility and survival via the mechanisms in (1) and (2), only.

To see the case (1), imagine two reproductive strategies, called *A* and *B*. Suppose that each has an age-dependent function *m*_0_, which is the fertility function as *N* → 0. Suppose also that fertility then decreases exponentially with *N* for both strategies at a rate *D*, i.e.,

$$\begin{array}{c} m_{A}\left(x,N\right)=m_{0,A}\left(x\right)e^{-DN}\;\;\;\;\;\text{and}\;\;\;\;\;m_{B}\left(x,N\right)=m_{0,B}\left(x\right)e^{-DN} \end{array}$$ (5)

*D* measures the strength of density dependence. As assumed in (1), the survival functions *l*_*A*_ and *l*_*B*_ are density-independent. Then the ratio *R*_*A*_/*R*_*B*_ for any *N* is

$$\begin{array}{c} \frac{R_{A}\left(N\right)}{R_{B}\left(N\right)}=\frac{\int_{x=0}^{\infty }l_{A}\left(x\right)m_{0,A}\left(x\right)e^{-DN}dx}{\int_{x=0}^{\infty }l_{B}\left(x\right)m_{0,B}\left(x\right)e^{-DN}dx}. \end{array}$$ (6)

The exponential terms, being independent of age, can be removed from both integrals and then canceled out, yielding

$$\begin{array}{c} \frac{R_{A}\left(N\right)}{R_{B}\left(N\right)}=\frac{\int_{x=0}^{\infty }l_{A}\left(x\right)m_{0,A}\left(x\right)dx}{\int_{x=0}^{\infty }l_{B}\left(x\right)m_{0,B}\left(x\right)dx}=\frac{R_{A}\left(0\right)}{R_{B}\left(0\right)}, \end{array}$$ (7)

which is independent of *N*. The math is essentially the same for cases (2) and (3).

It is easy to imagine plausible cases where these conditions do not hold. For example, suppose that the *D*’s are *not* equal for *A* and *B*—in other words, one strategy is more affected by crowding than the other. Then the exponential terms in Equation (6) would not cancel, allowing the possibility for the ordering of *R*’s to vary by *N*. Another possibility is to imagine a simple form of density-dependent survival: suppose the probability of survival *at each age* is affected by a simple exponential multiplier, similar to above. Because the probability of surviving to age *x* is the product of survival probabilities at each previous age, the exponential term now contains *x* in the exponent:

$$\begin{array}{c} l_{A}\left(x,N\right)=l_{0,A}\left(x\right)e^{-DNx}\;\;\;\;\;\text{and}\;\;\;\;\;l_{B}\left(x,N\right)=l_{0,B}\left(x\right)e^{-DNx}. \end{array}$$ (8)

If we suppose fertility is density independent, then the ratio *R*_*A*_/*R*_*B*_ is

$$\begin{array}{c} \frac{R_{A}\left(N\right)}{R_{B}\left(N\right)}=\frac{\int_{x=0}^{\infty }l_{0,A}\left(x\right)e^{-DNx}m_{A}\left(x\right)dx}{\int_{x=0}^{\infty }l_{0,B}\left(x\right)e^{-DNx}m_{B}\left(x\right)dx}. \end{array}$$ (9)

Now the term involving *N* is age dependent (i.e., it includes *x*), so it cannot be removed from the integral and canceled. The relative orderings of *R* will not necessarily be independent of *N*, and therefore we cannot use *R* to deduce the optimal strategy.

The three conditions noted above do not appear to be well appreciated, especially among anthropologists. There is no mention of these strict conditions by Kaplan et al. [1], for example. This is probably because life history theorists have not always been clear about demographic assumptions in the classic models. For example, Hawkes et al. [3] rely on Charnov’s *R*-based models, including a paper in which Charnov claims that *R* is “a Darwinian fitness measure appropriate for a nongrowing population,” and uses it as a maximand [19]. We have seen that this is incorrect in general. Fortunately, Charnov’s models frequently assume the second density-dependence case listed above (see, e.g., [20, 17]), for which *R* is appropriate. Unfortunately, this form of density dependence is not mentioned in the articles cited by [3]; it is not surprising, then, that they do not state restrictions themselves. The three conditions are also not noted influential life history textbooks [8, 10, 9].

The broad conclusion here is that one cannot study the evolution of a density-dependent population in general by maximizing a density-*independent* function of *R*. Further, the assumption of zero population growth alone does not justify *R* as a maximand. *R* is only appropriate under the specific forms of density dependence listed above. Whether these conditions are accurate for human populations is largely unknown; certainly they have not been justified by anthropologists.

## The coevolution of age at maturity and investment in survival

I now shift attention to the model of Kaplan et al. [1]. I review the assumptions and claims of the model, and then reanalyze it under various forms of population regulation. My goal is not to discredit Kaplan et al.’s hypothesis (which I think is promising), but to demonstrate that the results they derive fail under many forms of population regulation.

### The model

The model in Kaplan et al. ([1], p. 165) treats the coevolution of two life history characteristics: age at maturity and investment in survival. Individuals experience two life stages: a juvenile, pre-reproductive stage, and an adult, reproductive stage, which begins at age *t*. In both stages, individuals invest some proportion *λ* of available energy to mortality reduction. Among juveniles, the remaining energy is devoted to the development of embodied capital (growth and learning). Among adults, the remaining energy is devoted to reproduction. Only *t* and *λ* evolve in this model.

The instantaneous death rate, *μ*, is constant across ages for any given strategy. Since *λ* measures investment in mortality reduction, *μ* is a decreasing function of *λ*: strategies with high *λ* live longer. To allow for external effects on mortality, the parameter *θ* is introduced to quantify the extrinsic mortality risk. *μ* is an increasing function of *θ*.

Kaplan et al. do not provide fertility functions, but they do provide a term that represents the energy invested in fertility. I simply let this equal fertility outright, so that an *m* function is recovered. Thus, fertility at age *t* is equal to the embodied capital produced up to that point, multiplied by 1−*λ* (as the rest of the energy continues to be invested in survival). Let the embodied capital at age *t* be denoted by *P*(*t*,*λ*,*ε*). *P* is an increasing function of *t*: more time in maturity allows for more embodied capital. It is a decreasing function of *λ*, as this energy is lost to survival investment. *ε* is an ecological parameter that measures the ease of the environment with respect to fertility: all other things being equal, greater *ε* implies higher fertility. Finally, Kaplan et al. assume that energy production grows at an exponential rate *g* after maturity due to skills and knowledge acquired from experience during the reproductive stage. I translate this energy production directly to fertility.

### The claimed results

Kaplan et al. claim six general results with regard to evolution in *t* and *λ*. Let $\hat{t}$ and $\hat{\lambda }$ be the optimal values of age at maturity and investment in mortality reduction, respectively. Then Kaplan et al. claim

$$\begin{array}{c} \frac{\partial \hat{t}}{\partial \epsilon }>0\;\;\;\;\;\;\;\;\;\;\frac{\partial \hat{\lambda }}{\partial \epsilon }>0 \end{array}$$ (10)

$$\begin{array}{c} \frac{\partial \hat{t}}{\partial \theta }<0\;\;\;\;\;\;\;\;\;\;\frac{\partial \hat{\lambda }}{\partial \theta }<0 \end{array}$$ (11)

$$\begin{array}{c} \frac{\partial \hat{t}}{\partial g}>0\;\;\;\;\;\;\;\;\;\;\frac{\partial \hat{\lambda }}{\partial g}>0 \end{array}$$ (12)

Inequalities (10) say that, all other things being equal, niches more hospitable to reproduction favor later maturity and greater investment in mortality reduction. Inequalities (11) say that as extrinsic mortality increases, selection favors earlier maturity and lesser investment in mortality reduction. Inequalities (12) say that a greater growth rate of energy production (and therefore fertility) after maturity selects for later maturity and greater investment in mortality reduction.

Kaplan et al. emphasize not only the directional effects of the ecological parameters, but also the positive coevolutionary relationship between age at maturity and investment in survival. For each parameter change, they found that the age at maturity and the investment in survival always increased or decreased together (i.e., for each line, the derivatives have the same sign).

### Reanalysis

#### Forms of population regulation

The main problem with the original analysis in Kaplan et al. is a failure to specify the form of population regulation, precluding identification of the proper maximand. Instead, they claim zero population growth without specifying precisely how this occurs, and then assume that selection maximizes a density-independent *R*. To reanalyze the model properly, one must first specify forms of population regulation; I choose three simple cases here (although two produce identical outcomes, as seen just below). I generalize by allowing one case of nonzero population growth, as well as two forms of density dependence.

Consider three cases of population regulation:

1. Density-independent (exponential) growth
2. Density-dependent fertility
3. Density-dependent mortality

In case 1, all fertility and mortality rates are fixed quantities, independent of population density. Selection maximizes *r* here, whereas it maximizes $\hat{N}$ for all other cases. This would be reasonable if, over the evolutionary timescale of interest, human populations were largely regulated by density-independent factors.

In case 2, fertility across all ages and strategies depends on population size via the same multiplier. This is precisely the condition under which *R* happens to be maximized, as discussed above (see equations (5) and (6)). As shown there, I assume that *m*(*x*,*N*) = e^−*DN*^ *m*_0_(*x*), where *m*_0_ is the fertility under zero population density and *D* quantifies the extent to which fertility is adversely affected by population density. Survival rates are independent of density.

In case 3, the same form of density dependence applies to mortality for each age, rather than fertility. That is, *l*(*x*,*N*) = e^−*DNx*^ *l*_0_(*x*). Fertility is independent of density. This form, too, was mentioned in the previous section (equations (8) and (9)), but selection does not maximize *R* here.

Note that in both density-dependent cases, the *D* parameters are assumed to be fixed. That is, all strategies are affected by density equally. I assume this only for simplicity, as different strategies could realistically vary in their susceptibility to crowding. Fortunately, this assumption also leads to a useful simplification: *cases 1 and 3 favor the exact same strategy for *l* and *m**. This follows because *r* and $D\hat{N}$, which are respectively maximized in cases 1 and 3, take the same functional form in the Euler-Lotka equation, as is seen by comparing the integral of Equation (1) to those of (9). This simplification implies that we need only consider two cases: cases 1 and 3 together, and case 2. These simple differences in population regulation lead to very different optimal life history strategies.

#### Cases 1 and 3

Under density-independent population growth (case 1), we have the following demographic equations:

$$\begin{array}{c} l\left(x\right)=e^{-\mu (\lambda ,\theta )x} \end{array}$$ (13)

$$\begin{array}{c} m(x)=0\;\;\;\;\;\text{for}\;x<t \end{array}$$ (14)

$$\begin{array}{c} m\left(x\right)=\left(1-\lambda \right)P\left(t,\lambda ,\epsilon \right)e^{g(x-t)}\;\;\;\;\;\text{for}\;x\geq t \end{array}$$ (15)

Under density-dependent mortality (case 3), *l*(*x*) is multiplied by the term e^−*DNx*^ as described above. For *R* to remain finite, we require that *g* < *μ*. I assume this throughout, as do Kaplan et al. [1].

Under density-independence, a strategy’s growth rate is found by plugging the functions (13), (14), and (15) into Equation (1). Doing this, integrating, and simplifying produces

$$\begin{array}{c} 1=\frac{e^{-(\mu +r)t}\left(1-\lambda \right)P}{\mu +r-g}. \end{array}$$

Solving for *r* produces

$$\begin{array}{c} r=\frac{\text{W}[e^{-gt}\left(1-\lambda \right)P\left(t,\lambda ,\epsilon \right)t]}{t}+g-\mu \left(\lambda ,\theta \right). \end{array}$$ (16)

W is the product-log function: W[*z*] solves the equation *z* = *W*[*z*]*e* ^*W*[*z*]^. Though W is a complicated function, it is useful to know that *W*[*z*] > 0 for *z* > 0 and d*W*/d*z* > 0, i.e. the product-log is an increasing function of its argument.

An invading strategy will only invade if the *r* of the invading strategy is greater than that of the resident strategy. If mutant strategies both *t* and *λ* tend to differ from their resident values by a small amount, then the direction of evolution is predicted by the derivatives of *r* with respect to both strategy variables. Furthermore, any internal equilibrium in *t* and *λ* must satisfy the condition that both derivatives equal 0. Thus we search for optima via the simultaneous solution of d*r*/d*t* = 0 and d*r*/d*λ* = 0. Simplifying these gives us

$$0=\frac{\partial \log\left(P\left(t,\lambda ,\epsilon \right)\right)}{\partial t}-r-\mu \left(\lambda ,\theta \right)$$ (17)

$$0=-\frac{\partial \mu \left(\lambda ,\theta \right)}{\partial \lambda }\left(t+\frac{1}{r+\mu \left(\lambda ,\theta \right)-g}\right)+\frac{\partial \log\left(P\left(t,\lambda ,\epsilon \right)\right)}{\partial t}-\frac{1}{1-\lambda },$$ (18)

where *r* is given by Equation (16). (We must also check that the solution actually maximizes, rather than minimizes, *r*.) Note that these differ from equations (2) and (3) in Kaplan et al. [1] (reproduced below in (21) and (22)).

The analogous equations for the case of density-dependent mortality are found by replacing *r* in the above equations with $D\hat{N}$, subject to the constraint that $\hat{N}$ implies 0 population growth as in Equation (3). These two models favor the same strategy at equilibrium, so all further results apply to both.

Due to the complexity of these equations, I have been unable to derive any general results about parameter effects, leaving claims (10), (11), and (12) difficult to evaluate in general. To proceed I assume explicit functions for *P* and *μ* and seek numerical solutions. I assume the functions

$$\begin{array}{cc} P(t,\lambda ,\epsilon ) & =e^{\epsilon }\left(1-\lambda \right)\frac{\log(1+\alpha \beta t)}{\beta } \end{array}$$ (19)

$$\begin{array}{cc} \mu (\lambda ,\theta ) & =\mu_{0}e^{-\gamma \lambda }e^{\theta } \end{array}$$ (20)

Equation (19) implies that fertility at maturity is a diminishing-returns function of age at maturity. The parameter *α* measures the initial rate at which embodied capital is acquired during the juvenile period. *β* is the diminishing-returns parameter: with higher *β*, the more quickly returns to learning and growth diminish. In Equation (20), *γ* determines the rate at which investments in survival (*λ*) decrease mortality risk. The remaining parameters were defined above and satisfy the assumptions of Kaplan et al. [1].

**Fig 1:**
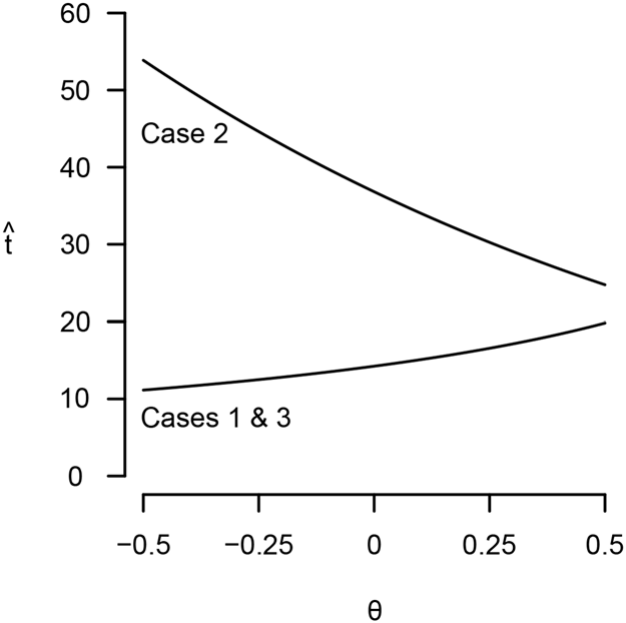
The optimal age at maturity, $\hat{t}$, may either decrease or increase with extrinsic mortality risk, *θ*, depending on the form of population regulation. *α* = 0.1, *β* = 0.2, *μ*_0_ = 1, *γ* = 5, *ε* = 0, *g* = 0.

**Fig 2:**
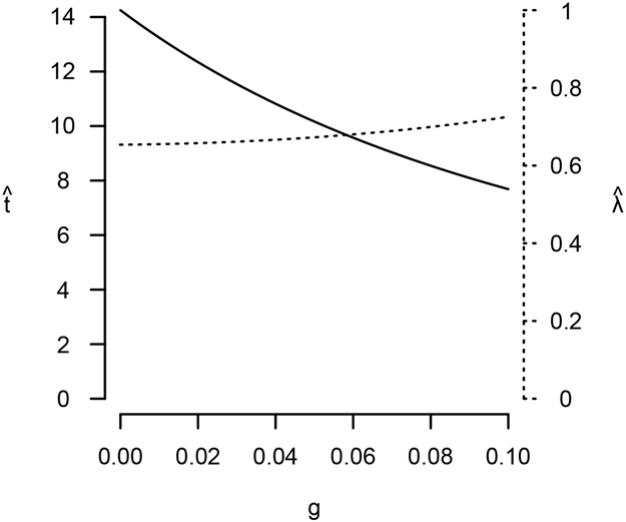
Under cases 1 and 3, *t* and *λ* evolve in different directions when *g* increases. The solid line depicts $\hat{t}$, while the dashed line depicts $\hat{\lambda }$. *α* = 0.1, *β* = 0.2, *μ*_0_ = 1, *γ* = 5, *ε* = 0, *θ* = 0.

Equations (19) and (20) can be substituted into Equations (17) and (18), which can then be solved numerically. While this procedure cannot prove general results, contradictions to most of Kaplan et al.’s claims readily arise (see Figs. 1 and 2). Under all parameter values numerically investigated (see supporting text), I find that, contrary to inequalities (10), $\hat{t}$ and $\hat{\lambda }$ both *decrease* as *ε* increases; niches more hospitable to reproduction prompt earlier maturity and lesser investment in survival. Contrary to inequalities (11), $\hat{t}$ and $\hat{\lambda }$ both *increase* with *θ*; niches with greater extrinsic mortality risk favor later maturity and greater investment in survival. Finally, while $\hat{\lambda }$ does increase with *g* as Kaplan et al. predicted, $\hat{t}$ decreases; niches in which production continues to grow in adulthood favor earlier maturity. This latter finding also shows that *λ* and *t* may not evolve in the same direction.

#### Case 2

Repeating the same process as above, but assuming that population density only affects fertility, we arrive at the equilibrium conditions

$$0=\frac{\partial \log\left(P\left(t,\lambda ,\epsilon \right)\right)}{\partial t}-\mu \left(\lambda ,\theta \right)$$ (21)

$$0=-\frac{\partial \mu \left(\lambda ,\theta \right)}{\partial \lambda }\left(t+\frac{1}{\mu \left(\lambda ,\theta \right)-g}\right)+\frac{\partial \log\left(P\left(t,\lambda ,\epsilon \right)\right)}{\partial \lambda }-\frac{1}{1-\lambda }$$ (22)

These are equivalent to equations (2) and (3) in Kaplan et al. [1]. This is expected because this form of density dependence maximizes *R* under zero population density.

Curiously, some of Kaplan et al.’s results do not hold in this case, suggesting that the authors made assumptions that were not printed in the paper. Again assuming functions (19) and (20), I first find that *ε* drops out entirely; the extrinsic costs of fertility have no effect on the equilibria. Second, numerical calculations show that *λ* evolves upward with *θ*, which is opposite to *t*. Finally, both *t* and *λ* evolve upward with *g*, as Kaplan et al. predict.

### What can we conclude?

Despite the small set of demographic conditions investigated here, my analysis quickly found contradictions to the general claims of Kaplan et al. [1]. More complicated forms of population regulation would probably produce still more widely varying outcomes. Thus any strong claims like inequalities (10), (11), and (12) seem unlikely to hold in general; there is probably *some* form of density-dependence for which almost any conclusion does not hold.

Still, to avoid leaving the reader with the unsatisfying message that “anything can happen,” I conducted a systematic search for general parameter effects (the supporting text describes the methods). I found two consistent effects: first, increasing *α*, the initial rate at which embodied capital grows, always favors earlier maturity and lesser investment in survival. Second, increasing *g*, the rate at which production (and, hence, fertility) grows after maturity, always favors greater investment in survival. Fortunately, these conclusions are compatible with Kaplan et al.’s general argument: if the transition to the intensive human foraging niche caused slower initial rates of growth and learning (due to the difficulty of acquiring complex skills), and allowed for greater production growth during adulthood, then this may well have contributed to the evolution of the human extended life history. Other ecological effects, unfortunately, remain ambiguous. For example, if the niche transition also implied higher extrinsic mortality due to the difficulty of acquiring resources, then this could have selected for either shorter or longer life histories, depending on the form of population regulation (Fig. 1).

## Directions for future research

Even my extended analysis of Kaplan et al.’s [1] model remains limited. I did not allow the strength of density dependence to vary across ages or strategies, effectively assuming that all individuals are equally affected by population density. The functional forms I chose for numerical evaluation were only one of an infinite number of possibilities. I ignored the potential problems of cyclic or chaotic population dynamics, as well as all of the difficulties introduced by frequency-dependence, separate sexes, realistic genetic structures, and environmental change. Theory exists for all these details, but it is doubtful that broad generalizations like (10), (11), and (12) would hold even under a limited range of realistic model assumptions.

The difficulty of finding such broad generalizations suggests that we need to shift our focus away from broad-scope evolutionary models and toward understanding how human populations have been regulated throughout our evolutionary history. Knowledge of these details would allow theorists to constrain model space to only the most realistic cases, leading to more pointed predictions. Studies of density-dependent effects in human populations may be necessary, but are surprisingly rare. I know only of the study by Wood and Smouse [21], which found evidence of density-dependent mortality among the very youngest and oldest age classes in a New Guinea population. Studies among large, nonhuman mammals also find that juvenile mortality is more sensitive to environmental stress than is adult mortality ([22, 23]; but see [24] for a case where adult mortality is driven strongly by predation). Adult fertility among nonhuman mammals also appears to be sensitive, although this is sometimes confounded with yearling survival, and may depend on the mother’s age [25]. That the effects on fertility and mortality depend on age suggest that the age-independent models treated in this paper cannot be taken too seriously. On the other hand, if it is true that density dependence acts mostly on fertility and infant survival, then case 2 may turn out to be the closest to reality. In any case, it is clear that both theoretical and empirical work is needed to advance our understanding of human life history evolution.

## Supporting Information

S1 File Methods for exploring parameter space. (PDF)
