# Supplementary material for: The Importance of Population Growth and Regulation in Human Life History Evolution
Source: PLoS One. 2015 Apr 1;10(4):e0119789. doi: 10.1371/journal.pone.0119789 (PMC4382128; doi:10.1371/journal.pone.0119789)
Supplement: S1 File — (PDF) [file pone.0119789.s001.pdf]

Supplementary to "The importance of population growth and regulation in human life history evolution"

Ryan Baldini*

February 4, 2015

Methods for exploring parameter space

The size of the parameter space precludes exhaustive exploration in this paper. I conducted a restricted search by varying only two parameters at a time, leaving the remaining parameters fixed at baseline values. This allows for identification of first-order interactions between parameters with respect to their effect on $\hat{t}$ and $\hat{\lambda}$. For each interaction (e.g. α and β), 100 values were uniformly selected from the range of both variables, producing a 100x100 matrix and therefore 10,000 separate model equilibria. Equilibria were numerically calculated using the rootSolve package in R.

Baseline values were $\alpha = 0.1$, $\beta = 0.2$, $\mu_0 = 0$, $\gamma = 5$, and $\epsilon = \theta = g = 0$. The parameters α and β were varied by plus and minus an order of magnitude (e.g. 0.01 to 1 for α). The remaining parameters involved exponential functions, so were varied over a smaller, additive range. γ ranged from 1 to 10; ϵ and θ ranged from -5 to 5; and g ranged from 0 to 1. Since variation in μ_0 has the same effect as variation in θ, μ_0 was not varied.

For model case 1 or 3, I discarded results for any parameter set where r or $\hat{N}$ was calculated to be less than 0, respectively. The condition was identical across the two cases, so the same results again apply to both. For model case 2, I discarded results for which $\hat{N} < 0$ or $\hat{N} = \infty$. The latter is equivalent to the condition that $g > \mu$, which was assumed to not hold in the text, for the same reason. The range of parameter values I used usually surpassed these limits, indicating that the parameter space was well-explored for first-order interactions

*Department of Anthropology. Stanford University, Stanford, CA 94305. ryanbaldini@gmail.com
